# Highlights of three metabolites HDL and reduction in blood pressure values after dietary fiber supplementation in overweight and obese normotensive women: a metabolomic study

**DOI:** 10.1007/s11306-023-02057-z

**Published:** 2023-11-17

**Authors:** Cássia Surama Oliveira da Silva, Mussara Gomes Cavalcanti Alves Monteiro, Carla Patricia Novaes dos Santos Fechine, Josean Fechine Tavares, Augusto Lopes Souto, Rafaella Cristhine Pordeus Luna, Flávia Cristina Fernandes Pimenta, Ana Herminia Andrade e Silva, Alcides da Silva Diniz, Celso Costa da Silva Júnior, Caio César Ferreira Alverga, Sócrates Golzio dos Santos, Darlene Camati Persuhn, Maria José de Carvalho Costa

**Affiliations:** 1 Postgraduate Program in Nutrition Sciences, Federal University of Paraíba, João, Pessoa 58059-900 Brazil; 2 Postgraduate Program in Public health, Federal University of Paraíba, João, Pessoa 58059-900 Brazil; 3 Department of Pharmaceutical Sciences, Federal University of Paraiba, João, Pessoa 58059-900 Brazil; 4 Department of Internal Medicine, Federal University of Paraiba, João, Pessoa 58059-900 Brazil; 5 Department of Statistics, Centre for Exact and Natural Sciences, Federal University of Paraiba, João, Pessoa 58059-900 Brazil; 6 Department of Nutrition, Health Sciences Center, Federal University of Pernambuco, Recife, 50670-901 Brazil

**Keywords:** Dietary fiber, Blood pressure, Nuclear magnetic resonance, Metabolome

## Abstract

**Introduction:**

The prevalence of hypertension and obesity are a worldwide concern.

**Objetives:**

Assess the metabolites profile after intervention with mixed dietary fiber in overweight and obese normotensive women.

**Methods:**

This is a randomized double blind placebo-controlled study. Through a simple randomization process, two groups were allocated, with eleven women (group 1) receiving 12 g of mixed dietary fiber and thirteen women (group 2) receiving 12 g of placebo (corn starch) for eight weeks. Anthropometric and biochemical tests and lifestyle were analyzed. As for evaluation metabolomics, used a ^1^H NMR. The data matrix generated 96 samples and 225 variables, which was exported in the ASCII format for the “The Unscrumbler” statistics software (version 9.7, CAMO Process).

**Results:**

After the intervention with mixed dietary fiber, significant differences were observed between the main types of metabolites, referring to the increase in the relative peak areas of in three HDL metabolites 4.94 ppm (0.0086*), HDL 1.28 ppm (0 .0337*), HDL 0.88 ppm (0.0224*) and an α-glucose metabolite 4.90 ppm (0.0106) and the reduction in systolic blood pressure (SBP) (0.0292*) of 7 mmHg in the reference range and in the placebo group there was a reduction in SBP (0.0118*) of 4 mmHg and of a choline metabolite 3.65 ppm (0.0266*), which does not call into question the validity of these results in the literature.

**Conclusion:**

The synergism of the functions of these statistically highlighted metabolites contributed to prevention the increase in SBP after fiber intervention in overweight and obese normotensive women.

**Supplementary information:**

The online version contains supplementary material available at 10.1007/s11306-023-02057-z.

## Introduction

Hypertension and obesity are a worldwide concern (Amin et al. 2021; Didaniele et al. 2021;  Kong et al. 2023), and the western lifestyle, particularly driven by food choices, among other habits, has been consistently associated with the prevalence of hypertension, obesity and cardiovascular diseases (CVD) (Arnett et al. 2019; Clemente-Suárez et al. 2023; Kaye et al. 2020; Pearson et al. 2021). The development of innovative studies to better understand how to prevent these morbidities, such as metabolomics, is increasingly pertinent.

As a preventive strategy, dietary fiber intake stands out; however, there is still no consensus regarding its recommendation in guidelines on prevention and treatment of cardiovascular (CV) risk factors with a focus on arterial hypertension (Whelton et al. 2018), although it has already been associated with the prevention of CV disorders, with improvements in blood glucose and insulin resistance, aiding in weight loss and blood pressure (BP) reduction (Barber et al. 2020; Fechine et al. 2021; Reynolds et al. 2019; Reynolds et al. 2020; ). Therefore, as an early strategy to prevent these morbidities, we hypothesize that intervention with mixed dietary fiber for eight weeks in in overweight and obese normotensive women can attenuate the increase in blood pressure and prevent the increase in overweight and obesity.

Metabolomics has been used to assess metabolic disorders associated with the development of hypertension and obesity ( He et al. 2020; Htun et al. 2021; Ke et al. 2018; Pasanta et al. 2019; Onuh & Aliani, 2020; Rangel-huerta et al. 2019), but there is no intervention study with mixed dietary fiber and prevention of hypertension and obesity in overweight and obese normotensive women.

The specific metabolomic profile for overweight normotensive individuals of both sexes has been studied, showing metabolites such as amino acids, phosphatidylcholines, lysophosphatidylcholines (Auguet et al. 2023; Bagheri et al. 2019; Htun et al. 2021), high-density lipoproteins (HDL-C) (Pasanta et al. 2019) and that these changes can be delayed or attenuated by increasing dietary fiber consumption (Mayengbam et al. 2019).

In this sense, the present study aimed to evaluate statistically highlighted metabolites after the intervention with mixed dietary fiber in overweight and obese normotensive women in the prevention of BP increase within the reference limits, in comparison with the placebo group, an unprecedented topic in the literature consulted.

## Methods

### Study design and sample characteristics

This is a randomized, double-blind, placebo-controlled intervention study in which participants were recruited at the Blood Center of the municipality of João Pessoa - Paraíba (PB) - Brazil from March to December 2018. They were invited and then signed the Informed Consent Form (TCLE). The project was submitted to the Research Ethics Committee of the Health Sciences Center (CCS) of the Federal University of Paraíba (UFPB), approved under CAAE number 64573917.4.0000.5188, and registered in the Brazilian Registry of Clinical Trials under number TRIAL: RBR-2PH4F9.

Sample calculation was carried out based on the effect of the fiber found on pressure values in a pilot study. The study adopted 95% reliability (Z α/2 = 1.96), a β error of 20% [with power of 80%] (Z β = 0.84). Subsequently, the standard deviation of this distribution was estimated; a fiber effect difference of 12 mmHg was considered and n of at least 11 women per group was found. Participants were randomly distributed into two groups based on medical diagnosis regarding blood pressure values, exclusion criteria (hypertension, diabetes, liver failure, congestive heart failure (CHF), and renal failure), and type of intervention.

The initial number of participants was 30. However, there was a sample loss due to inclusion criteria (n = 4) and withdrawal stemming from the occurrence of adverse effects as a result of the use of fiber (colic and diarrhea n = 2). This resulted in a final tally of 11 participants in group one (G1) and 13 participants in group two (G2).

This study consisted of 24 overweight and obese normotensive women aged 20–50 years, with those in G1 using mixed dietary fiber (12 g/day) and G2 using placebo (cornstarch, 12 g/day). The intervention period lasted eight weeks for both groups.

The mixed dietary fiber was composed of soluble (7 g) and insoluble (5 g) fibers. Soluble fibers were composed of Gum Guar (4g; lot ALL 0605354), Nutraflora (FOS; 1g; lot Galena (CIQ): 1505006204), Psyllium (2g; scientific name Plantago ovata; manufacturer batch: 949/2013) and insoluble fibers (5g; Microcrystalline Cellulose 101–5g, batch 14116094A, manufacturing batch C1404014, formula C6nH10n + 205n = 1). Mixed dietary fiber and placebo were manufactured at a third-party pharmacy.

Regarding inclusion criteria, they were overweight and obese normotensive women with systolic blood pressure (SBP) ≤ 130 mmHg and diastolic blood pressure (DBP) ≤ 80 mmHg, age between 20 and 50 years, and a body mass index (BMI) above 25 up to 35 kilograms per square meter (Kg/m^2^). The exclusion criteria were as follows: hypertension, diabetes, liver failure, congestive heart failure (grades 3 and 4) and renal failure with creatinine values greater than 3.0 mg/dL.

The selected women who agreed to participate in the intervention study intended to kept their eating habits and level of physical activity stable for four weeks before the start of the intervention and communicated that they did not intend to change them throughout the study.

### Analysis of biochemical parameters

For biochemical analyses, blood collection was performed by a nurse who used three different sterile tubes for venipuncture: tube 1 (with anticoagulant K3 EDTA-ethylenediamine tetraacetic acid), tube 2 (with anticoagulant sodium fluoride), and tube 3 (with clot activator). Samples in tubes 2 and 3 were immediately centrifuged to obtain plasma and serum, respectively. These were submitted for analysis less than 2 h after collection.

Pre-and post-intervention analyses of the lipid profile were performed and included total cholesterol (TC), triglycerides (TG), high-density lipoproteins (HDL-C) and low-density lipoproteins (LDL-C), and fasting glucose and ultrasensitive C-reactive protein (us-CRP).

For the determination of lipid fractions of total cholesterol (TC), HDL-C and TG were measured using enzymatic assay (Allain et al. 1974; Bucolo & David, 1973; ). TC and TG (enzymatic method - Trinder) were measured in serum aliquots in an automatic analyzer using a Labtest kit. The desirable reference value for HDL-C is > 40mg/dL (Magalhaes, 2017).

Fasting plasma glucose was measured using the enzymatic method (Cooper, 1973), considering the following reference values: ≤ 99 mg/dL (normal), 100–125 mg/dL (pre-diabetes) and ≥ 126 mg/dL (diabetes) (American diabetes association, 2017).

### Blood pressure evaluation

For the blood pressure measurement, criteria established by the Brazilian Society of Cardiology, in its Hypertension Guideline (Sbc, 2010), were adopted. These criteria consist of taking three blood pressure (BP) measurements with an interval of at least one minute between them using OMRON HEM-742INT equipment. Women were at rest (5 minutes) and in a sitting position with their feet flat on the ground and their arms resting on the table (Unger et al. 2020).

### Nutritional evaluation

BMI was classified according to the criteria recommended by the World Health Organization (WHO, 2000) for adults aged 20–59 years with overweight (25.0–29.9 kg/m2) and obesity (30.0–39.9 kg/m2) were used. Weight and height measurements were performed in triplicate, respecting the average of three values (Kac et al. 2007).

### Food consumption evaluation

To assess food consumption, a 24-hour recall (R24h) was used. It was carried out by professional nutritionists and applied twice to each participant on the first day of the study and after eight weeks; that is, it was applied in two moments. To help with the answers, an album of drawings of the average food portions was used (Ascuitt et al. 2005), whose reference was the use of a frequency questionnaire validated for the population of women from the northeast region of Brazil (Lima et al. 2008) to assess the usual food consumption of the sample. A questionnaire developed for this purpose was applied (Costa et al. 2013).

Recalls were entered into the Virtual Nutri Plus software. For the food consumption analysis, to estimate habitual food consumption and correct intrapersonal variance, the online Multiple Source Method (MSM) was used, available on the website (https://msm.dife.de/tps/msm/), prepared by the European Prospective Investigation into Cancer and Nutrition (EPIC) (Msm, 2012).

### Metabolomic profile

1H nuclear magnetic resonance (NMR) was used to analyze the metabolomic profile. It was carried out at the Multiuser Laboratory of Characterization and Analysis (LMCA) at UFPB in a Bruker model spectrometer operating at 400 MHz. The pulse sequence used to obtain the 1H spectra was CPMG PR1D, number of scans: 128, receiver gain: 58.8, acquisition time: 4.08s, spectral size: 64k and temperature: 30 °C and were performed in triplicates.

Initially, 5 ml of blood was collected in polypropylene tubes with EDTA in the early morning, during fasting. After collection, it was centrifuged at 3000 xg for 10 min at 4 °C to isolate the serum. Serum was transferred to a sterilized tube and stored at − 80 °C. It was thawed for use. 300 µL of serum was collected and 300 µL of phosphate buffer was added as a reference standard. Vortex homogenization was performed for 30 s, immediately after centrifiguation at 14000 x g for 10 min at 4 °C. After centrifugation, 60 µL of deuterated water was added. Then, analysis was performed in the NMR apparatus. The supernatant was transferred to a 400 MHz NMR tube in a 5 mm tube. The pulse sequence used to obtain data was CPMG-BRUKER and analyses were performed in triplicate.

The obtained spectra were analyzed using the TopSpin 4.1.1 software and chemical shifts were expressed in ppm. Metabolites were identified by comparing chemical shifts with chemical shifts of authentic samples available in the Human Metabolome Database (HMDB). Spectra were referenced by tetramethylsilane (TMS), whose total range chosen for analysis was between 0.1 and 9 ppm, with binnings of 0.02 ppm. This generated a data matrix of 96 samples and 225 variables, which was exported in the ASCII format for the “The Unscrumbler” statistics software (version 9.7, CAMO Process). Data were not normalized by area, and peaks referring to TMS and water were discarded. With these procedures, it was possible to differentiate phases before and after intervention with mixed dietary fiber.

### Statistical analyses

All analyses were performed using the R software version 4.1.1, free and available at https://www.r-project.org/. The significance level adopted throughout the analysis was 5%. Initially, the sample was characterized before the intervention, by group, using the descriptive measures of mean and standard deviation for quantitative variables, as well as simple frequency and percentage for qualitative variables.

For quantitative variables, the Shapiro-Wilk normality test was initially used to test the null hypothesis that the variable follows an approximately normal distribution versus the alternative hypothesis that the variable does not follow an approximately normal distribution. For variables considered approximately normal, Student's t-test was used for independent samples to verify whether there was a difference between groups for that variable. If the variable normality hypothesis was rejected, the Wilcoxon Mann–Whitney test was applied. As for qualitative variables, the chi-square test was initially used to test the null hypothesis that there is no association between the group and the variable classes versus the alternative hypothesis that there is an association between the group and the variable classes. For variables with any expected frequency of less than five, Fisher's exact association test was used to test the same hypothesis.

To compare the metabolomic profile of women in each group, before and after the intervention, for each metabolite and each individual, the triplicate mean was calculated to obtain a single value for each individual. To compare the metabolomic profile in each group, the Shapiro-Wilk normality test was initially used to test the null hypothesis that the metabolite follows an approximately normal distribution versus the alternative hypothesis that the metabolite does not follow an approximately normal distribution. For metabolites considered approximately normal, Student's t-test was used for dependent samples to verify whether there was a difference before and after intervention for that metabolite. If the hypothesis of metabolite normality was rejected, the Wilcoxon test was applied.

Data obtained from NMR were processed by the MestreNova software (version 6.02, MestreLab Research S.L) and chemical shifts were expressed in ppm using the Principal Component Analysis (PCA) statistical tool.

## Results

The sample characteristics in relation to clinical, demographic, and lifestyle variables are presented in Table 1.

**Table 1:** Clinical and demographic characteristics and comparison of variables by group before intervention (N = 24) of the sample of overweight and obese normotensive women recruited at the Blood Center of João Pessoa, PB, Brazil

Variables	G1 (n = 11)	G2 (n = 13)	*p*
Age (years)	36.09 ± 14.25	40.15 ± 8.39	0.4183^1^
Weight (kg)	83.30 ± 14.70	75.16 ± 8.64	0.1264^1^
Height (m)	1.59 ± 0.05	1.61 ± 0.05	0.4004^1^
BMI (kg/m²)	29.08 ± 3.08	29.08 ± 3.08	0.1191^2^
Overweight	5 (45.45%)	7 (53.85%)	1.0000^3^
Obesity	6 (54.55%)	6 (46.15%)	
Total cholesterol (mg/dL)	202.20 ± 36.53	189.15 ± 22.58	0.7064^2^
TG (mg/dL)	114.09 ± 42.03	111.38 ± 39.91	0.8738^1^
HDL-C	59.09 ± 14.60	63.85 ± 13.17	0.4155^1^
LDL-C	119.91 ± 32.58	104.15 ± 24.31	0.2023^1^
VLDL-C	23.09 ± 6.31	21.23 ± 5.21	0.7229^1^
SBP (mmHg)	119.54 ± 10.34	112.61 ± 10.02	0.0626^2^
DBP (mmHg)	76.73 ± 6.99	77.69 ± 7.33	0.8769^1^
Fasting blood glucose (mg/dL)	94.54 ± 9.76	94.85 ± 11.80	0.9768^1^
Use of medications	0 (0.00%)	0 (0.00%)	0.6831^1^
Schooling (years of study)	14.18 ± 4.02	14.77 ± 3.03	0.7707^1^
(less than 14 years)	4 (36.36%)	5 (38.46%)	1.0000^4^
(more than 14 years)	7 (63.64%)	8 (61.54%)	
Physical activity	5 (45.45%)	3 (30.00%)	0.3905^4^
Alcohol consumption	1 (9.09%)	0 (0.00%)	0.4583^4^
Family income in dollars	1293.26 ± 889.95	1561.89 ± 1284.74	0.7720^2^

^*^Significant result ^1^Student’s t-test for independent samples ^2^Wilcoxon-Mann-Whitney test ^3^Chi-square test ^4^Fisher's exact test

The BP values and the lipid profiles were compared before and after the intervention, which showed a significant difference in relation to SBP both G1 (SBP before = 119.54 ± 10.34 mmHg and SBP after = 112.92 ± 6, 41 (p = 0.0228)) and G2 (SBP before = 112.61 ± 10.02 mmHg and SBP after = 108.31 ± 10.03 (p = 0.0118)), thus demonstrating a reduction in SBP after the intervention, in both groups, results not demonstrated. As for the lipid profile (TC, TG, HDL-C and LDL-C) before and after the intervention, there was no significant difference both G1 and G2, results not demonstrated.

Regarding the usual food consumption, no difference was observed before and after intervention regarding calories (K cal), carbohydrates (g) and (%), protein (g) and (%), lipids (g) and (%), total fiber (g) and (%), potassium (g) and (%), and sodium (g) and (%), (results not shown).

**Fig. 1:**
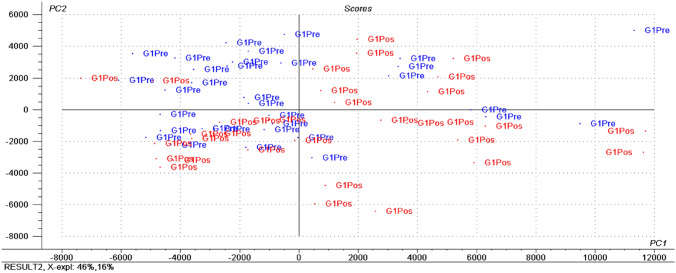
1H NMR multivariate data analysis extracted from serum analysis of overweight and obese normotensive women before and after mixed dietary fiber intervention. Statistical tool - Principal Component Analysis (PCA). Score Plot. G1 Pre (Blue group: pre-intervention); G1 Pos (Red group: post-intervention). The G1 Pos group was differentiated due to the influence of peaks related to HDL and sugar metabolites

Figure 1, 2 shows the most prominent metabolites before and after interventions for G1.

**Fig. 2:**
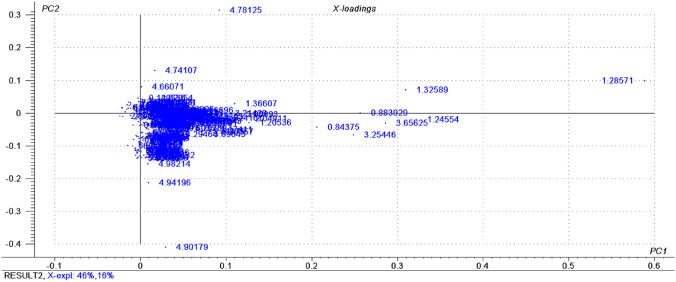
1H NMR multivariate data analysis extracted from serum analysis of overweight and obese normotensive women before and after mixed dietary fiber intervention. Statistical tool - Principal Component Analysis (PCA). Loadings Plot. Representation of all detected metabolites and their spatial influence in relation to the samples distributed in the Score Plot

The following highlighted metabolites, in relation to Fig. 2, were 4.94 (HDL); 4.90 (α-glucose); 4.74 (β glucose); 3.65 (choline); 3.25 (choline); 1.36 (lactate); 1.32 (HDL); 1.28 (HDL); 1.24 (HDL); 0.88 (HDL); and 0.84 (HDL).

**Fig. 3:**
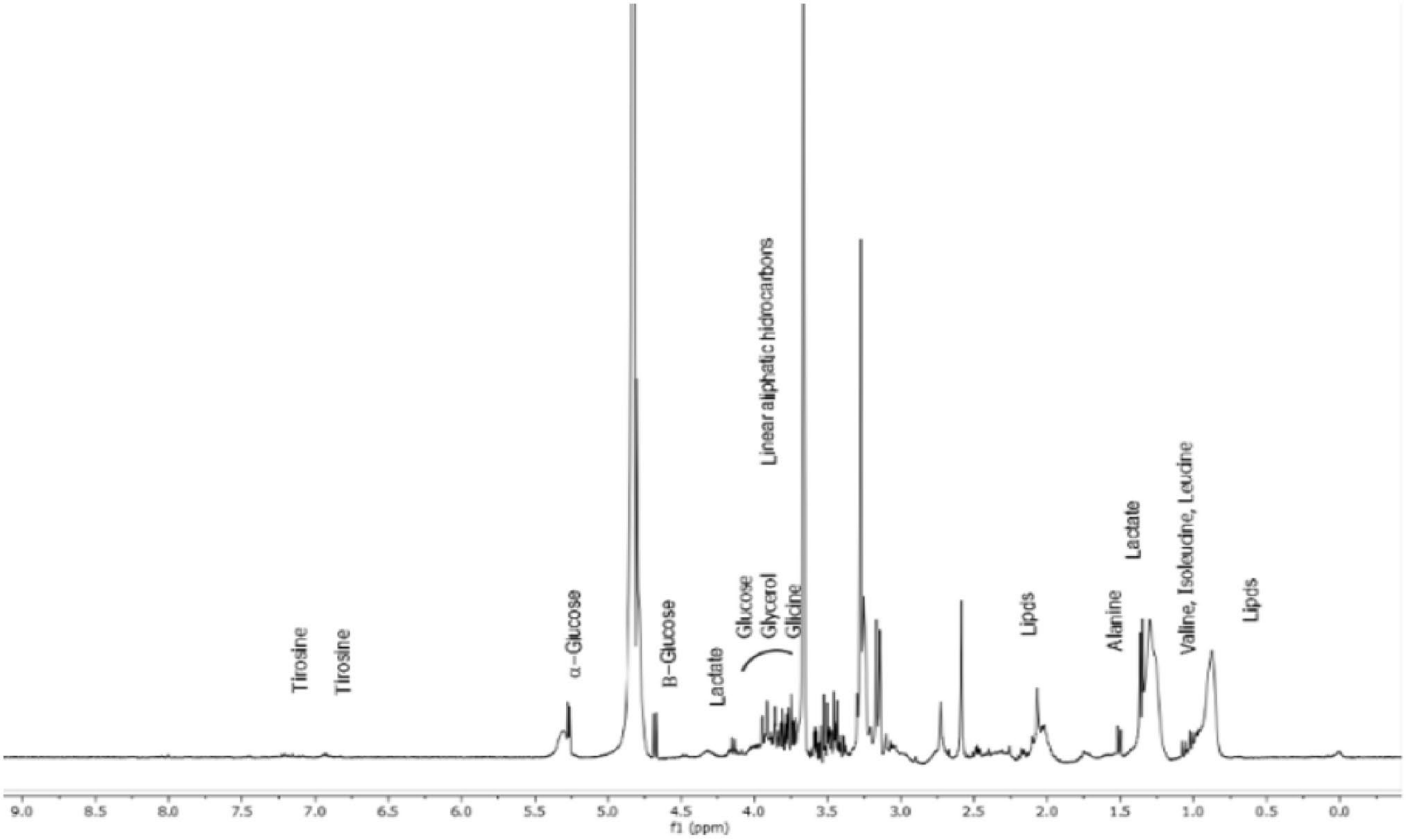
1D Carr Purcell Meiboom Gill (CPMG) 1H NMR spectrum representative of serum metabolites. * PPM (chemical shift)

The 1D Carr Purcell Meiboom Gill (CPMG) 1H NMR spectrum representative of normotensive women after mixed dietary fiber supplementation is shown in Fig. 3.

Considering the comparison between the metabolomic profile of overweight and obese normotensive women, before and after intervention with mixed dietary fibers (Table 2), several significant results were observed; these were the metabolites that stood out based on the peak area (= PPM-chemical shift): *4.94 ppm (HDL) *1.28 ppm (HDL); *0.88 ppm (HDL) and *4.90 ppm (α glucose), in which an increase in the values of HDL and α-glucose metabolites were observed.

**Table 2:** Comparison between study variables regarding metabolite values before and after intervention with mixed dietary fibers (G1 = 11) of overweight and obese normotensive women attending the Blood Center of João Pessoa, PB, Brazil

Metabolites	Signal/RMN	Before	After	*p*
PPM**	PPM**			
HDL	4.94	106.01 ± 287.21	687.83 ± 625.37	0.0086^1^*
Lactate	1.36	917.08 ± 382.52	1147.03 ± 424.69	0.0537^1^
HDL	1.32	2424.30 ± 1025.88	3015.45 ± 1261.60	0.0513^1^
HDL	1.28	4246.54 ± 1979.22	5464.34 ± 2442.02	0.0337^1^*
HDL	1.24	2695.03 ± 1158.99	3346.91 ± 1113.33	0.0651^1^
HDL	0.88	2142.83 ± 852.43	2728.74 ± 912.14	0.0224^1^*
HDL	0.84	2018.62 ± 724.85	2382.40 ± 655.87	0.2402^2^
α-glucose	4.90	307.80 ± 315.68	1559.44 ± 1264.57	0.0106^1^*
β-glucose	4.74	339.16 ± 363.77	252.10 ± 422.92	0.5935^1^
Choline	3.65	3248.36 ± 1526.69	2956.27 ± 1160.30	0.5195^2^
Choline	3.25	2761.38 ± 956.07	2820.75 ± 828.97	0.8000^1^

^*^Significant result ^1^Student's t-test for dependent samples ^2^Wilcoxon test

The highlighted metabolites, in relation to G2, were 4.90 ppm (α-glucose); 4.74 ppm (β-glucose); 3.65 ppm (choline); 3.25 ppm (choline); 1.36 ppm (lactate); 1.32 ppm (HDL); 1.28 ppm (HDL); 1.24 ppm (HDL); 0.88 ppm (HDL); and 0.84 ppm (HDL).

Considering the comparison between the metabolomic profile of overweight and obese normotensive women before and after intervention with placebo, significant results were observed; these were the metabolites that stood out based on the peak area (= PPM- chemical shift): *3.65 ppm (choline) a reduction in choline values were observed.

## Discussion

It was identified for the first time, after data analysis using NMR and comparison with spectra from the HMDB database, significant highlights referring to the unique increase in the relative peaks areas of three different HDL metabolites (4.94 ppm; 1.28 ppm; 0. 88 ppm) and an α-glucose (4.90 ppm) and the reduction in the SBP, in overweight and obese normotensive women, after supplementation with mixed dietary fibers, and also identifying a reduction in the SBP and a reduction in choline metabolite (3.65 ppm) in the placebo group.

Researchers observed that normotensive individuals with obesity have reduced levels of HDL-C (Bellot et al. 2023; Rezaee et al. 2022; Zhu et al. 2022). Furthermore, in the context of obesity, the morphology and function of adipose tissue are modified, which can affect HDL-C functioning (Wang & Peng, 2011; Zhang et al. 2019).

Soluble fibers, also called viscous fibers, may reduce blood cholesterol through of fecal bile salts excretion and delayed and reduced absorption, but the effect of fiber on increasing HDL-C is not conclusive (Lioy et al. 2023), and in the present study it contained 60% soluble fiber. According to Fechine et al. (2021) observed that, in hypertensive women with excesso weight after intervention with dietary fiber, the levels of HDL-C and HDL metabolites (4.94 ppm; 1.28 ppm; 1.24 ppm; 1.32 ppm; 0.88 ppm) increased and reduced SBP and DBP. Thus, the application of NMR-based metabolomics in blood analysis provides information on whole-body metabolic processes (Bartel et al. 2015), offering opportunities to clarify how fiber intake affects the metabolism of obese individuals (He & Bertram, 2022). Studies on metabolomics and obesity without intervention in perimenopausal women (Ding et al. 2021), as well as in premenopausal women (Wiklund et al. 2014) and young men and women (Pasanta et al. 2019), highlighted lipid, glucose, amino acid, and organic acid metabolites. Under these health conditions, metabolites grouped by PCA in these studies are similar to those of the present study.

Based on the analyzes performed by Reynolds et al. (2019), in several clinical studies using conventional methods, it was observed that dietary fiber intake reduced body weight, blood cholesterol and systolic blood pressure (SBP). Despite all these mechanisms, they are still not enough to explain the protective effect of dietary fiber intake in preventing the increase in BP and body weight (Bartolomeaus et al. 2019; Du et al. 2021; Waddell & Orfila, 2022). Therefore, more studies with innovative technologies are needed.

In this sense, this is the first molecular study in which a reduction in SBP and increase of the relative peak areas different HDL and α-glucose metabolites were observed in overweight and obese normotensive women who consumed mixed dietary fibers.

High serum HDL-C, considering the upper limit of the reference range, is associated with good HDL quality and functionality (Cho, 2022), it was observed in the present study that no group, after the intervention, presented HDL-C < 40 mg/dL (low HDL-C), and among individuals in G1 9% (1 individual, HDL-C pre = 24 mg/dL; HDL-C post = 56 mg/dL) and in G2 8% (1 individual, HDL-C pre = 92 mg/dL; HDL-C post = 78 mg/dL). Therefore, change in HDL-C values, according to the results found in the present study, described above, referring to the increase or reduction of HDL-C, is not always directly proportional to the value before the intervention, probably reflecting in several interfering factors on these values, such as lifestyle and genetics ( León-Reyes et al. 2023; Wang et al. 2023).

The quality of HDL-C refers to the morphology of HDL-C, such as size, shape, and number of particles and depends on the composition of HDL, in terms of apolipoproteins (apoA-I, apoA-II, apoC-III, serum amyloid A and α -synuclein), cholesterol, and triglycerides, and is also associated with the extent of HDL modification, such as glycation and oxidation, resulting in apoA-I multimerization, and aggregation leading to amyloidogenesis and often determining HDL functionality, which depends on activity of the attached antioxidant enzyme, such as the paraoxonase and cholesterol efflux activity (Cho, 2022; Cho, 2019a, 2019b).

Conventional HDL functionality is regression, referent the removal of cholesterol from atherosclerotic lesions, and the removal of oxidized species in low-density lipoproteins (LDL) (Cho, 2019b). Based on the above, future molecular studies should investigate the quality and functionality of HDL metabolites, considering that in the present study there was a reduction in SBP, and a significant increase in all the relative areas of the peaks of HDL metabolites, after intervention with mixed dietary fiber, leading to the reflection that possibly the increase in these metabolites involves a reduction in SBP, in the present case series.

As for the increase in the relative peak area of α-glucose metabolite (4.90 ppm), in conventional studies, according to Samra and Anderson (2007), fiber reduces postprandial glucose levels in healthy individuals. However, in the present study, with a molecular focus, after intervention with fibers, increase in the relative peak area of α-glucose metabolite (4.90 ppm) was observed, which together with β-glucose represents the total glucose (Pasanta et al. 2019), and which, according to Htun et al. (2021) is high in obese young adults without intervention. As for the results of the present study, regarding glucose, it should be reevaluated and new research, using NMR methods without water suppression.

Therefore, further studies are needed to justify the increase found in the present study, that is, to examine whether this increase in the relative peak area of α-glucose metabolite was due to the expected metabolic signature in obesity (Htun et al. 2021) and whether the amount of fiber consumed in the intervention was not enough to lower the relative peak area value of α-glucose metabolite. As for the results of the present study, regarding glucose, it should be reevaluated and new research, using NMR methods without water suppression.

It is also highlighted in the present study that before the intervention, SBP was higher in G1 than in G2, but not statistically, as well as the amount of fiber usually consumed, before the study, was higher in G2 (20.63 ± 16.08) than in G1 (16.90 ± 5.71), but not statistically, which may have influenced the reduction in SBP in G2.

Therefore, we can say, in the present study, that there was a reduction in SBP in overweight and obese normotensive women after ditary fiber supplementation, corroborating with the study of Fechine et al. (2021), in overweight and obese hypertensive women, when observing that HDL metabolites (4.94 ppm; 1.28 ppm; 0.88 ppm) (unpublished results in the consulted literature), also contributed to this reduction both in normotensive women, in the present study, as well as in overweight and obese hypertensive women after ditary fiber supplementation (Fechine et al. 2021). In the present study, likely, the increase in the three HDL metabolites, will contribute to the increase in HDL-C, with a longer intervention time.

In the present study, regarding the significant effects of corn starch a placebo, reducing the values of SBP and choline metabolite, which may also likely have its origin in morbidity, in overweight and obese normotensive women, there is a need for emergency clarifying researches on these effects, in molecular nutrition. In this sense, Kaptchuk et al. (2008), when analyzing variables that interfere with the effects found related to placebo such as corn starch, and different placebo, recommend carrying out studies on the mechanisms of these effects, highlighting that the effect of the placebos used also occurred in other studies (Akiyama et al. 2023; Nasser et al. 2023; Sandra et al. 2023), and according to Kwon et al. (2020), an exploratory study in postmenopausal women with hypercholesterolemia, which aimed to investigate the effect of Korean Red Ginseng on cholesterol metabolites, it was observed that in the placebo group that received corn starch there were also changes in the metabolite 7-hydroxycholesterol (7-OHC), although significantly lower than in the intervention group. Highlighting that according to Kaptchuk et al. (2008), regarding the expected effect, the placebo administration method is as important as the administration of the substance to be tested itself. Furthermore, according to Iolascon and Moretti. (2021) the research participants show improvements simply because they are part of them. Therefore, the results found with placebo in the present study do not call into question the validity of the results.

Strengths include the importance of the theme in preventing the development of hypertension in overweight and obese normotensive women, an unprecedented theme in literature, and status as the first study to observe an increase in the relative peaks area different HDL metabolites.

As for the study’s limitations, the population under study consisted only of women. The effect of dietary fiber on BP reduction in men and different age groups should be confirmed in the future. The results encourage further studies including genetic aspects associated with metabolomics.

## Conclusions

Intervention with mixed dietary fibers reduction in SBP values, increased the relative peak areas of HDL contributed to body weight maintenance in overweight and obese normotensive women.

Therefore, the results of the present study provided comprehensive information on contributions to the metabolic signature based on the consumption of mixed dietary fibers of the sample analyzed in this study and facilitated the determination that the synergism between the functions of these metabolites, can be targets for the prevention of hypertension.

### Supplementary information

Below is the link to the electronic supplementary material.

Supplementary material 1 (PPTX 53.1 kb)

## Data Availability

The data presented in this study are available on request from the corresponding author
