# Supplementary material for: Highlights of three metabolites HDL and reduction in blood pressure values after dietary fiber supplementation in overweight and obese normotensive women: a metabolomic study
Source: Metabolomics. 2023 Nov 17;19(12):95. doi: 10.1007/s11306-023-02057-z (PMC10656339; doi:10.1007/s11306-023-02057-z)
Supplement: Supplementary file 1 — Supplementary material 1 (PPTX 53.1 kb) [file 11306_2023_2057_MOESM1_ESM.pptx]

## Slide 1
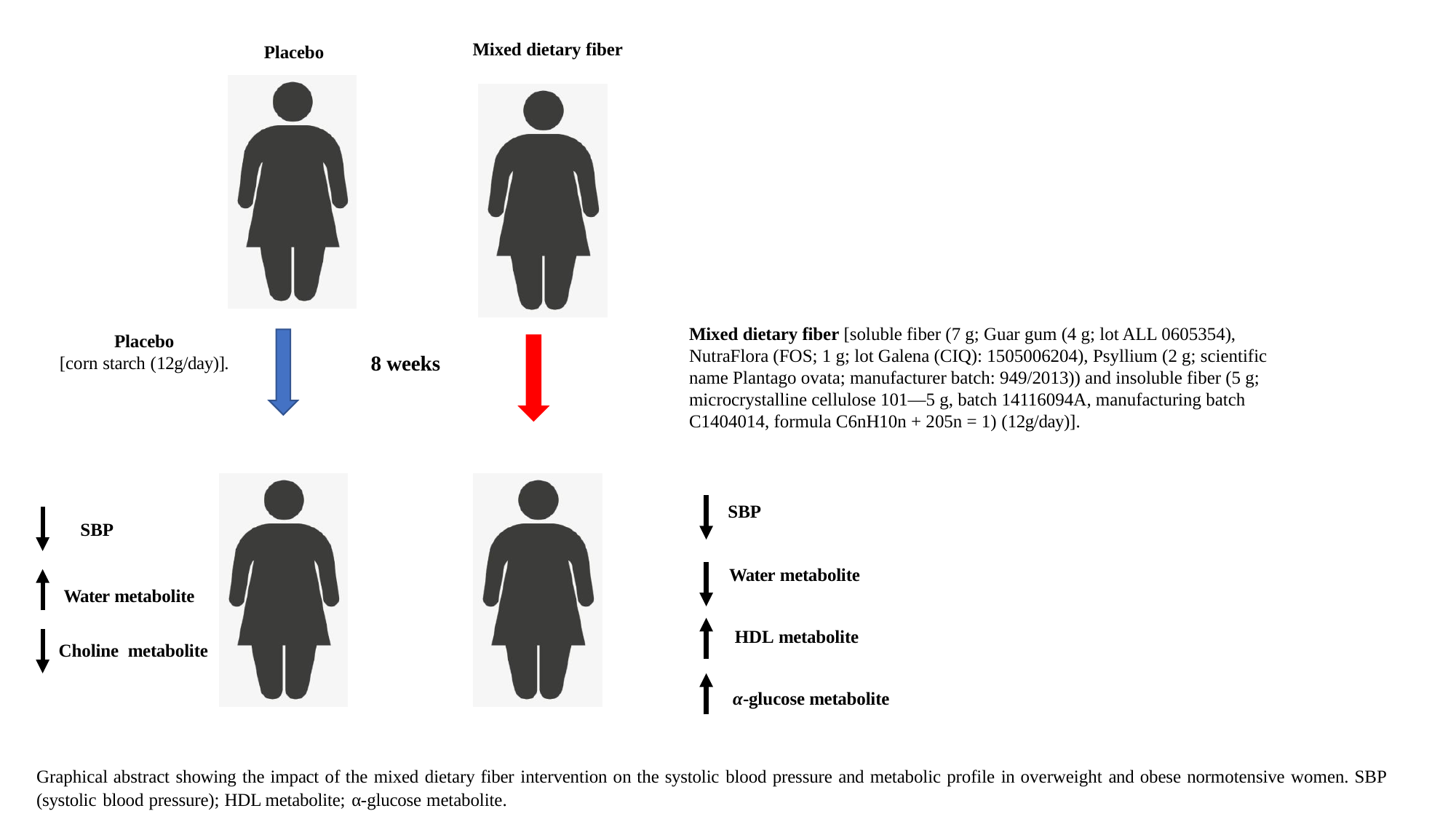

Mixed dietary fiber
Placebo
Mixed dietary fiber [soluble fiber (7 g; Guar gum (4 g; lot ALL 0605354), NutraFlora (FOS; 1 g; lot Galena (CIQ): 1505006204), Psyllium (2 g; scientific name Plantago ovata; manufacturer batch: 949/2013)) and insoluble fiber (5 g; microcrystalline cellulose 101—5 g, batch 14116094A, manufacturing batch C1404014, formula C6nH10n + 205n = 1) (12g/day)].
Placebo
[corn starch (12g/day)].
8 weeks
SBP
SBP
Water metabolite
Water metabolite
HDL metabolite
Choline metabolite
α-glucose metabolite
Graphical abstract showing the impact of the mixed dietary fiber intervention on the systolic blood pressure and metabolic profile in overweight and obese normotensive women. SBP (systolic blood pressure); HDL metabolite; α-glucose metabolite.
